# Remarks upon the Nature of Gout, and the Action of Remedies in That Disease

**Published:** 1815-01

**Authors:** Richard Walker

**Affiliations:** Oxford


*For the Medical and Physical Journal.*

*Remarks upon the Nature of Gout, and the Action of Remedies in that Disease;* by Mr. R. WALKER, of Oxford.

PERCEIVING that the subject of Gout is at this time much and zealously agitated, I beg leave to anticipate, in some degree, my intended paper on this subject by the following observations.

With respect to gout, I am enabled by the most convincing experience to assert, that I have ascertained to demonstration the following facts, viz. 1st, that it consists in a morbid tumour, or acrimony generated in the habit. 2dly, That the method of cure, or prevention, consists either in cutting off the source of the disease, or constantly carrying it off as fast as generated, by a convenient channel or outlet. 3dly, That, though much may be done towards cutting off or diminishing the disposition for generating this disease, by medicine and regimen, yet these means, separately or conjointly, act very imperfectly and partially in obviating or carrying off the humour or matter of gout; and, moreover, weaken and debilitate the habit of body. 4thly.

4thly, That no drug or medicine in nature possesses the power of acting specifically upon gout; but that the effects produced depend merely and entirely upon some increased secretion occasioned by such medicine; and likewise incidentally and transiently by a temporary disturbance or derangement in the habit, thus putting aside as it were, or suspending, for the time present, an impending or commenced paroxysm of gout. 5thly, That, whatever drug or composition possesses the power of effecting the required secretions, together with a sedative or anodyne quality, will be a remedy equal in efficacy to the eau medicinale, or any imitation of it. The secretions I principally allude to, are, primarily, by the skin, or perspiration; and, secondarily, by the bowels.* 6thly, That the means I have pointed out, or alluded to, is an effectual and complete remedy, inasmuch as it completely obviates any future attack of gout, or if, in some instances, it should fail in this, it renders such attacks much less frequent, and, beyond all comparison, milder. 7thly, That, in order to produce the full effect to be expected from this remedy, and to regulate it so as to be free from any annoyance to the patient, requires an additional knowledge, not ordinarily known to any professional person, the particular directions for which I shall give hereafter. 8thly and lastly, That it is much to be regretted that the same good effects are not to be produced by occasionally swallowing a medicine; but which, long and adequate experience has convinced me, is never to be expected, such a remedy most probably not existing in nature.

With respect to the data upon which this opinion is founded, I shall have occasion to explain hereafter, in a paper entitled "On the Nature of Diseases, and the extent of power or efficacy of Medicine over them," in which I shall attempt to define or show the actual boundary of the power of medicine upon diseases—in short, in other words, to give the *ne plus ultra* of the medical art, founded upon fact, and reconcileable by reason.

I have at this time two patients, in whom this plan of treatment has been pursued,—one in whom the gouty diathesis showed itself most decidedly at a little past the age of thirty, by a repetition, during several years, of most violent

* It may be said, and I am ready to admit, that the *eau medicinale*, or any medicine possessing similar powers, might act successfully by its strong sedative powers alone, as we know happens from a somewhat similar cause, by sudden danger, alarms, &c. but what will be the state of the patient if a system of this kind is to be persevered in.

attacks of *genuine gout.* About the age of thirty-five, the remedy was applied, and most attentively managed; the paroxysms became immediately, or from that time, less frequent, and milder; and for several years past have entirely left him, except an incidental slight attack, of short duration, in one foot, scarcely occasioning limping, and this perhaps to be accounted for from inattention.

He lives, with respect to regimen, &c. in his ordinary way, which is by no means of a restrictive nature, and enjoys the most perfect health in every respect.

The other person has lately commenced the same plan, with a most striking and remarkable effect of a similar nature.

I have witnessed the effect of the eau medicinale, and, without adverting to the violent derangement it produces in the constitution of some persons, certainly should not recommend or administer it to any person, unless with a view of putting aside for the time an impending paroxysm, or, diminishing the violence of it if commenced; and I well know that even this effect from it is precarious, and by no means to be relied on; and, moreover, I have known it exhibited with extraordinary precaution, and according to the direction of the proprietor, produce subsequent debility and derangement of the constitution to such a degree, as to be long remembered and complained of by the persons who took it.

Whether the humour or essence of gout be of a peculiar nature, or *sui generis,* as we term it, or whether it be analogous to that species of humour ordinarily denominated scorbutic, &c. I do not know, nor is it material; but this I know, that an impending or commenced fit of gout is occasionally completely obviated, or carried off, by a determination of the humour to another part, commonly the surface, resembling a *scorbutic affection,* so called, and sometimes in the character of *true erysipelas*; and I well know, likewise, that the means I have alluded to, is a remedy both for *scorbutic acrimony* (to which, when it exists in the habit in a violent or virulent degree, I have assigned the name of *scorbutic scrophula**) and *gout.*

Could the same thing be effected by a *medicine,* it would be an important and valuable discovery indeed, but this I may venture to declare is never to be expected.

* This constitutional affection, though ordinarily considered of the same nature with *king's-evil,* or *genuine scrophula,* differs from that so much as to require a very different mode of treatment, and for which the plan above recommended is not applicable.

Here,

Here then, in effect, is a safe and certain remedy for gout, hitherto one of the great *opprobria medicorum;* with respect to the application of it, it rests with the patient to judge whether the remedy is more tolerable than the disease.

*Oxford;* RICHARD WALKER.

*Nov.* 4th, 1814.

